# Is there an association between non-alcoholic fatty liver disease and cognitive function? A systematic review

**DOI:** 10.1186/s12877-021-02721-w

**Published:** 2022-01-11

**Authors:** Elena S. George, Surbhi Sood, Robin M. Daly, Sze-Yen Tan

**Affiliations:** School of Exercise and Nutrition Sciences, Institute for Physical Activity and Nutrition (IPAN), Deakin University, Geelong, Victoria 3220 Australia

**Keywords:** NAFLD, NAFLD, NASH, Cirrhosis, Cognition, Cognitive function, Cognitive impairment

## Abstract

**Background:**

Non-alcoholic fatty liver disease (NAFLD) is represented as the most common liver disease worldwide. NAFLD is associated with metabolic risk factors underpinned by insulin resistance, inflammation and endothelial dysfunction, leading to extrahepatic changes in central nervous diseases such as cognitive impairment, Alzheimer’s disease and dementia. The aim of the review is to explore the association between NAFLD and cognitive function.

**Methods:**

Using the PRISMA guidelines, a systematic electronic literature search was conducted in four databases: MEDLINE, PsychINFO, Embase and CINAHL from inception until March 2021. Neuropsychological tests utilised within each study were grouped into relevant cognitive domains including ‘general cognition’, ‘reasoning’, ‘mental speed, attention and psychomotor speed’, ‘memory and learning’, ‘language’, ‘visuospatial perception’ and ‘ideas, abstraction, figural creations and mental flexibility’.

**Results:**

Eleven observational studies that involved 7978 participants with a mean age of 51 years were included. Those with NAFLD had poor cognitive performance in three cognitive domains, including ‘general cognition’, ‘mental speed, attention and psychomotor speed’, and ‘ideas, abstraction, figural creations and mental flexibility’.

**Conclusion:**

The observed results from the 11 included studies showed that NAFLD was associated with lower cognitive performance across several domains. However, studies conducted to date are limited to observational designs and are heterogeneous with varying diagnostic tools used to assess cognitive function.

**Trial registration:**

PROSPERO Registration: CRD42020161640.

## Background

Non-alcoholic fatty liver disease (NAFLD) is recognised as the most prevalent liver disease affecting approximately 30% of adults in Australia with similar, high rates in the United States [1–4]. The burden of NAFLD continues to rise significantly in Australia with current estimates of 5.5 million cases in 2019, with expectations of seven million cases by 2030 [1, 5]. NAFLD is defined as a spectrum of diseases related to hepatic fat deposition, ranging from non-alcoholic fatty liver (NAFL) (simple steatosis) to non-alcoholic steatohepatitis (NASH), which can progress to increased fibrosis, cirrhosis and hepatocellular carcinoma [3, 6]. Progression from NAFLD to NASH is often described using the “two hit” hypothesis. The “first hit” consists of lipid accumulation of fatty acids, increasing susceptibility of hepatocytes to secondary insults such as oxidative stress, insulin resistance and over production and release of pro-inflammatory cytokines. This can lead to the “second hit” which promotes steatohepatitis, chronic inflammation and fibrosis [7]. NAFLD occurs in the absence of excessive alcohol consumption and is associated with a range of common chronic disease risk factors such as insulin resistance, hypertension, obesity and visceral fat accumulation, and dyslipidaemia [1, 6]. Such risk factors are known to be elucidated by inflammation and oxidative stress, which also play a role in extrahepatic diseases, including central nervous system diseases such as mild cognitive impairment, Alzheimer’s disease, and dementia [8–10].

The global number of individuals living with dementia is 50 million [11], with the prevalence of cognitive impairment and dementia rising and estimated to increase amongst older adults (60 years and above) to approximately 2 billion by 2050, accounting for 22% of the world’s population [12]. Cognitive function encompasses multiple mental abilities and skills in reasoning, perception, memory, verbal and mathematical ability and problem solving [10, 13, 14]. Cognitive impairments have been associated with reduced ability to perform complex tasks such as driving and work-related activities leading to impaired quality of life and in more serious cases, premature mortality [15]. Cardiovascular disease (CVD), type 2 diabetes (T2DM) and metabolic syndrome (MetS) frequently co-exist with NAFLD and are also considered risk factors for cognitive decline [10] and dementia which are increased with ageing [16–18]. Individuals with NAFLD have high rates of metabolic syndrome components including dyslipidaemia, hypertension, abdominal obesity and insulin resistance, and there is also accumulating evidence that individuals with NAFLD have an increased risk of carotid atherosclerosis and carotid intima media thickness; all of which have been reported to contribute towards cognitive impairment [19, 20].

Previous cross-sectional and case-control studies have found that NAFLD is associated with poorer cognitive function across a number of cognitive domains as assessed using numerous common psychometric tests [21–23]. Studies in participants with NAFLD and hepatic encephalopathy have reported that they have lower brain volume [21], inflammation and hyperammonemia [24], all of which are associated with cognitive impairment. Despite the known link between NAFLD and various cardiometabolic-related diseases and the underlying mechanisms which drive these chronic diseases as well as cognitive decline, to date there has been no published systematic review summarising the relationship between NAFLD and cognitive impairment. Thus, the aim of this review was to systematically search the literature to explore the association between NAFLD and cognitive function.

## Methods

All methodology related to the analysis was specified prior to the literature search and detailed in a protocol registered with PROSPERO (CRD42020161640).

### Search strategy

The review adhered to the Preferred Reporting Items for Systematic Reviews and Meta-Analyses (PRISMA) guidelines [25], including independent execution of literature search and bias assessment, completed by author SS. MEDLINE, PsychINFO, Embase and CINAHL electronic databases were searched from inception until March 2021. The search terms were: (Cognit* or “Processing speed” or “mini mental state examination” or MMSE; Neuropsych* or Neurocognit* or Metacognit* or Recall or Memory or “Executive function” or “Verbal Fluency” or “Reaction time”) AND (“NAFLD” or “NASH” or “Cirrhosis” or “Non-alcoholic fatty liver” or “Nonalcoholic fatty liver” or “Non-alcoholic steatosis” or “Nonalcoholic steatosis”).

### Eligibility criteria

Studies of all designs were included if they were in English language, conducted in humans, included adults aged 18 years and over with NAFLD or at risk of NAFLD (as deemed in each paper where NAFLD was an outcome) and assessed cognitive function in individuals with NAFLD. Studies were excluded if they were review articles, abstract only, or included participants with mental health and neurodegenerative diseases such as Alzheimer’s disease, Parkinson’s Disease (PD) and PD-related disorders.

### Selection process

**Fig. 1:**
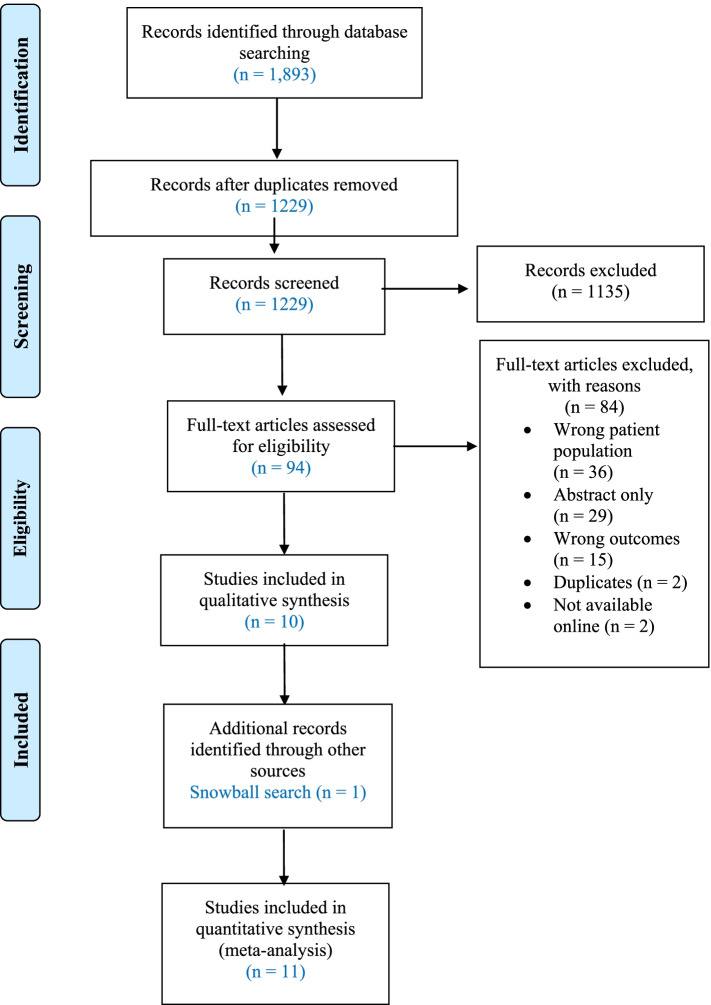
PRISMA

Title and abstract screening was carried out by one researcher according to the predefined protocol, and duplicates and articles which did not meet the eligibility criteria were excluded. Full text screening was conducted independently by two researchers (SS, SYT), and where there were any conflicts these were resolved by a third researcher (ESG). All articles included from the full-text screen were included in this systematic review. The search process is outlined in the PRISMA flowchart in Fig. 1.

### Data extraction and grouping

Data was extracted from 11 studies by one researcher and then re-checked by a second researcher. Data extraction included the following: author, year published, study design, length, population characteristics, presence of co-morbidities, the measurement methods for cognitive function and NAFLD, associations between NAFLD and cognitive function, and any other relevant outcomes (e.g. body fat, visceral fat, CVD risk factors). These findings are shown in Table 1. Due to the wide range of cognitive tests available and identified across the 11 studies, the cognitive tests were grouped into the following seven categories (general cognition, reasoning, mental speed and attention, memory and learning, language, visuospatial perception, ideas, abstraction, figural creations and mental flexibility) as described by Goodwill et al. [26]. Grouping was carried out by two researchers.

**Table 1:** Extraction table of included studies assessing the association between NAFLD and cognition^a^

Author, Year	Country	Study design; Length	Sample size	NAFLD mean age, sex (n females, n males)	Risk factors/co-morbidities	Cognitive tests	NAFLD diagnosis	Findings^b^
Weinstein et al. (2018) [27]	United States	Cross-sectional, population-based sampling survey; NHANES Survey data from 2011 to 2014	1102 participants 239 NAFLD participants	Mean NAFLD age: 68.6 years	NAFLD - BMI 35.0 kg/m2 Obesity: 92.5% Insulin resistance: 62.1% HTN: 76.1% High cholesterol: 82.2% MetS: 58.5% Control – BMI 25.00 kg/m2 Obesity: 19.4% Insulin resistance: 17.5% HTN: 61.2% High cholesterol: 74.2% MetS: 11.1%	**(1) The Word Learning subset** - used to assess immediate and delayed learning ability **(2) The Animal Fluency Test -** categorical verbal fluency **(3) The digit symbol substitution -** involves processing speed, sustained and working memory	Fatty liver index score ≥ 60 Spanish.	**(1) The Word Learning subset** – Immediate verbal memory: NAFLD only (SD: 20.5 ± 0.4) vs the control group (SD: 20.0 ± 0.3) Delayed verbal memory: NAFLD only group (SD: 6.6 ± 0.2) vs the control group (6.4 ± 0.1) **(2) AFT –** Verbal fluency: NAFLD only (18.6 ± 0.3) vs control group (18.3 ± 0.4) **(3) DSST –** Processing speed, sustained attention, working memory: NAFLD (55.9 ± 1.05) vs control group (53.6 ± 1.2)
Celikbilek et al. (2018) [22]	Turkey	Cross-sectional study	143 participants 70 NAFLD participants	Mean NAFLD age: 46.9 years, F (NAFLD): 41, M (NAFLD): 29	NAFLD – T2DM: 30% HTN: 20% Hyperlipidaemia: 7.1% MetS: 48.6% Control – T2DM: 4.1% HTN: 2.7% Hyperlipidaemia: 1.4% MetS: 34.3%	**(1) Montreal Cognitive Assessment** *Turkish version*. (a) visuospatial abilities - clock-drawing task and three-dimensional cube-copying task, (b) Memory - delayed recall (c) Executive functioning - Trail Making B task, a phonemic fluency task, and a two-item verbal abstraction task, (d) Attention - a serial subtraction task and digits forward and backward tasks, (e) Language - three-item confrontation naming task, repetition of two syntactically complex sentences (f) orientation - orientation to time and place is also evaluated	Abdominal ultrasonography	**(1) MoCA-TR -** scores significantly lower in NAFLD group in comparison to control group (P < 0.001) (a) Visuospatial abilities: *P* < 0.05 (b) Memory: *P* = 0.16 (c) Executive functioning: *P* < 0.05 (d) Attention: *P* = 0.56 (e) Language: *P* = 0.89 (f) Orientation: *P* = 0.29
Takahashi et al. (2017) [28]	Japan	Cross-sectional study	39 participants 24 NAFLD participants	Mean NAFLD age: 54 years	NAFLD – T2DM: 41.6% Dyslipidaemia: 58.3% HTN: 29.2% Control – Not reported	**(1) A verbal fluency task (VFT)**	Asia-Pacific Working Party guidelines for the assessment and management of NAFLD. Ultrasonography (in the absence of other causes of chronic liver disease e.g., hepatitis C antibody negative, hepatitis B surface antigen negative, and alcohol consumption < 20 g/day)	**(1) VFT –** Number of words during VFT was significantly higher in controls compared to the NAFLD: *p* < 0.032 *Number of words during VFT NAFLD: 12* *Number of words during VFT controls: 14*
Filipovic et al. (2018) [21]	Serbia	Case-control study	76 participants 40 NAFLD participants	Mean NAFLD age: 47.9 years, M (NAFLD): 22, F (NAFLD): 18	NAFLD – T2DM: 30% HTN: 80% Obese: 50% MetS: 85% Control – T2DM: 25% HTN: 44% Obese: 11% MetS: 22%	(**1) Montreal Cognitive Assessment test** *Serbian version*. (a) alternating connections, (b) vasoconstrictive abilities - draw a cube and a clock in 11:10 position of clock hands, (c) memory - numbers repeated in the same and reverse order, (d) attention - tap whenever you hear a letter A, serial subtraction of 7, starting with a hundred (e) sentence repeating and verbal fluency.	Abdominal ultrasonography, Sonographic evaluation (US) of hepatic steatosis	(**1) MOCA-SR –** The cognitive status was lower in NAFLD compared to control: OR 0.096, 95% CI 0.032–0.289, and p < 0.001
Elliott et al. (2013) [23]	UK (Europe)	Cohort, Prospective follow-up over 3 years	431 participants 224 NAFLD participant	Mean NAFLD age: 59 years, F (NAFLD): 101, M (NAFLD): 123	Not reported	**(1) Cognitive Failures Questionnaire (CFQ) -** measures memory, attention, concentration, forgetfulness, word finding abilities and confusion.	Liver Serum Biochemistry - ALT, ALP, ALB, Bilirubin.	**(1) CFQ –** Cognitive symptoms CFQ associated with worse functional ability in NAFLD compared to controls: r = 0.4, *p* < 0.0001
Weinstein et al. (2019) [29]	United States	Population-based, multi-generational study (The Framingham Heart Study) Cross-sectional study	1287 participants 378 NAFLD participants	Mean NAFLD age: 61.1 years, F (NAFLD): 157, M (NAFLD): 221	NAFLD – BMI: 31.0 T2DM: 20.5% HTN (stage 1): 59.3% Controls – BMI: 26.8 T2DM: 6.9% HTN (stage 1): 38.2%	**(1) The Wechsler Memory Scale** - verbal and visual memory **(2) Time to complete trail-making B minute time to complete trail-making A test (TrB-TrA) -** executive function **(3) The similarities test (SIM) -** abstract reasoning skills **(4) Hooper visual organization test (HVOT) -** visual perception.	Multi-detector CT with 8-slice, A calibration phantom (Image Analysis, Lexington, KY) with a water equivalent compound - three areas from the liver and one from an external phantom were measured (NAFLD was defined as having a liver/phantom ratio ≤ 0.33).	**(1) The Wechsler Memory Scale -** *p* = 0.610 **(2) TrB-TrA –** *p* = 0.418 **(3) SIM –** *p* = 0.746 **(4) HVOT –** *p* = 0.528 *No significant association of NAFLD with cognitive measures identified.* Participants with NAFLD with advanced fibrosis was associated with poorer performance on the TrB-TrA (*P* = 0.028) and SIM test (*P* = 0.009)
Tarter et al. (1984) [30]	United States	Case-control study	40 participants 30 NAFLD participants	Mean NAFLD age: 40.9 years	Not reported	**(1) Peabody picture intelligence test** – verbal intelligence. **(2) Raven’s progressive matrices** - nonverbal intelligence. **(3) Digit span forward**. **(4) Digit span backward**. **(5) Weschler Memory Scale):** (a) Logical memory (b) Figural memory, (c) Paired associates, (d) Suprasan. **(6) Perceptual-Motor:** (a) Finger tapping, (b) Purdue Pegboard, (c) Stary tracing, (d) Symbol digit modalities. **(7) Spatial:** (a) Block design, (b) Tactual performance, (c) Trail making. **(8) Language:** (a) Fluency, (b) Confrontation naming, (c) Responsive naming **(9) The Token Test** -comprehensive capacity	Hepatic diagnosis - clinical, biochemical, serological confirmed by liver biopsy	**(1) Peabody picture intelligence test –** no difference **(2) Raven’s progressive matrices –** no difference **(3) Digit span forward –** no difference **(4) Digit span backward –** no difference **(5) Weschler Memory Scale) –** no difference **(6) Perceptual-Motor –** (a) Finger tapping: no difference (b) Purdue Pegboard: *p* < 0.01 (c) Stary tracing: no difference (d) Symbol digit modalities: p < 0.01 **(7) Spatial –** (a) Block design: *p* < 0.06 (b) Tactual performance: *p* < 0.05 (c) Trail making: p < 0.05 **(8) Language –** (a) Fluency: no difference (b) Confrontation naming: no difference (c) Responsive naming: no difference **(9) The Token Test –** no difference
Tarter et al. (1987) [31]	United States	Case-control study	46 participants 23 NAFLD participants	Mean NAFLD age: 37.2 years, M (NAFLD): 11, F (NAFLD): 12	Not reported	**(1) Digit Span Test** - immediate memory **(2) Supraspan Learning Test -** the number of trials required accurately to repeat a string of digits that is one digit longer than the digits forward score. **(3) Benton Visual Retention Test (BVRT)** - short term visual **(4) Rey-Osterreith Complex Figure Test** - incidental learning ability **(5) Brown-Peterson Test** - measures the rate of decay of information from short-term memory.	Serological tests, clinical history and physical examinations, and was confirmed by a percutaneous liver biopsy.	**(1) Digit Span Test –** no difference **(2) Supraspan Learning Test –** p < 0.05 **(3) BVRT –** p < 0.0001 **(4) Rey-Osterreith Complex Figure Test** – no difference **(5) Brown-Peterson Test** – no difference
Seo et al. (2016) [32]	United States	Cross-sectional study (NHANES III - survey)	4472 participants 874 NAFLD participant	Mean NAFLD age: 40.9 years	NAFLD – T2DM: 13.6% HTN: 36.3% Hypercholesterolaemia: 30.4% Stroke: 1.5% Controls – T2DM: 5.2% HTN: 17.5% Hypercholesterolestelemia: 22.2% Stroke: 0.5%	**(1) The Simple Reaction Time Test (SRTT) -** measures response time, visual-motor speed **(2) The Symbol Digit Substitution Test (SDST) -** assesses visual attention and coding ability **(3) The Serial Digit Learning Test (SDLT) -** test learning, recall, and concentration.	Gallbladder examination by ultrasound with a Toshiba SSA-90A machine with a 3.75 and 5.0 MHz transducer. Liver enzymes (ALT, AST) were assayed with Hitachi 737 automated multichannel chemistry analyser. NAFLD was defined as moderate/severe steatosis as deter- mined by ultrasound.	**(1) SRTT –** B = 7.827, 95% CI 0.975 to 14.679 **(2) SDST –** B = 0.110, 95% CI 0.004 to 0.216 **(3) SDLT –** B = 0.880, 95% CI 0.317 to 1.443 *Participants with NAFLD had lower performances on SRTT, SDST and SDLT*
Felipo et al. (2012) [24]	Spain (Europe)	Case control study	179 participants 29 NAFLD participant	Mean NAFLD age: 45 years, F (NAFLD): 24, M (NAFLD): 5	Not reported	**(1) The Digit Symbol test (DST)** – mental speed **(2) The Number Connection Test A (NCT-A)** – mental speed **(3) The Number Connection Test B (NCT-B)** – mental speed **(4) The Serial Dotting Test** – mental speed **(5) The line tracing test (LTT)** – visuospatial	Liver biopsy obtained during histological study.	**(1) DST** – no difference in NAFLD, cirrhosis or NASH **(2) NCT-A** – no difference in NAFLD, impaired in patients with NASH and cirrhosis (*P* < 0.001) **(3) NCT-B** – no difference in NAFLD, impaired in NASH and cirrhosis (*P* < 0.001) **(4) The Serial Dotting Test** – no difference in NAFLD, impaired in cirrhosis (*P* < 0.001) and NASH (*P* < 0.01) **(5) LTT** – no difference in NAFLD, impaired in NASH and cirrhosis (P < 0.001)
Tuttolomondo et al. (2018) [33]	Italy (Europe)	Case control study	163 participants 80 NAFLD participants	Mean NAFLD age: 53.7 years	NAFLD – BMI: 28.7 HTN: 35.0% T2DM: 38.8% Dyslipidaemia: 18.8% Previous CVD: 3.75% Controls – BMI: 25.3 HTN: 15.7% T2DM: 8.4% Dyslipidaemia: 16.9% Previous CVD: 0	**(1) Mini-mental state examination (MMSE)**	Increased serum levels of ALT for at least 6 months and alcohol consumption of < 20 g/day in the previous year. Liver ultrasound finding of steatosis and a liver stiffness value > 6 kPa. Liver biopsy for NAFLD. Hepatic steatosis was defined by detection of Bright Liver Echo pattern.	**(1) MMSE** – NAFLD had a lower mean MMSE score when compared to controls (26.9 ± 1.6 vs. 28.0 ± 1.36; *p* = 0.005)

^a^*Abbreviations*: *AFT* animal fluency test, *ALT* alanine aminotransferase, *ALP* alkaline phosphatase, *AST* aspartate aminotransferase, *BMI* body mass index, *BVRT* Benton visual retention test, *CFQ* Cognitive Failures Questionnaire, *DSST* The digit symbol substitution test, *DST* digit symbol test, *HVOT* hooper visual organisation test, *HTN* hypertension, *LTT* line tracing test, *MoCA-TR* Montreal cognitive assessment Turkish version, *MoCA-SR* Montreal cognitive assessment Serbian version, *MCI* mild cognitive impairment, *MetS* metabolic syndrome, *NAFLD* non-alcoholic fatty liver disease, *NASH* non-alcoholic steatosis, *NCTA* number connection test A, *NCTB* number connection test B, *SIM* similarities test, *SRRT* simple reaction time test, *SDST* symbol digit substitution test, *SDT* serial dotting test, *SDLT* serial digit learning test, *VFT* verbal fluency task

^b^Significant effect (*p* < 0.05); no effect (*p* > 0.05); *CI* confidence interval, *OR* odds ratio, *RR* relative risk

### Quality assessment and risk of bias

The quality of the included papers and risk of bias was assessed independently using The Academy of Nutrition and Dietetics Evidence Analysis Library Quality Criteria Checklist **(**Table 2**)** [34]. This checklist consists of an evaluation of studies’ relevance (four questions) and validity (ten questions). Based on these criteria, the researcher assigned each article a quality rating of positive, neutral or negative (+, Ø, −).

**Table 2:** Critical appraisal of the 11 studies with the use of Quality Criteria Checklist^a^

	Celikbilek, 2018 [22]	Elliott, 2013 [23]	Felipo, 2012 [24]	Filipovic, 2018 [21]	Seo, 2016 [32]	Takahashi, 2017 [28]	Tarter, 1984 [30]	Tarter, 1987 [31]	Tuttolomondo, 2018 [33]	Weinstein, 2018 [27]	Weinstein, 2019 [29]
**Relevance Questions**											
1. Would implementing the studied intervention or procedure (if found successful) result in improved outcomes for the patients/clients/population group?	NA	NA	NA	NA	NA	NA	NA	NA	NA	NA	NA
2. Did the authors study an outcome (dependent variable) or topic that the patients/clients/population group would care about?	Y	Y	Y	Y	Y	Y	Y	Y	Y	Y	Y
3. Is the focus of the intervention or procedure (independent variable) or topic of study a common issue of concern to dietetics practice?	Y	Y	Y	Y	Y	Y	Y	Y	Y	Y	Y
4. Is the intervention or procedure feasible?	Y	Y	Y	Y	Y	Y	Y	Y	Y	Y	Y
**Validity Questions**											
1. Was the research question clearly stated?	Y	Y	Y	Y	Y	Y	Y	Y	Y	Y	Y
2. Was the selection of study subjects/patients free from bias?	Y	Y	Y	Y	Y	Y	N	N	Y	Y	Y
3. Were study groups comparable or was an appropriate reference standard used?	Y	N	Unclear	Y	Y	Y	N	Y	Y	Y	Y
4. Were methods of handling losses from the original sample (withdrawals) described?	Y	N	NA	Y	N	N	N	N	NA	NA	Y
5. Was blinding used to prevent introduction of bias?	Y	Y	Y	Y	Y	Y	Unclear	Y	Y	Y	Y
6. Was the intervention/treatment regimen/exposure factor, procedure, process or product of interest, and any comparison(s) described in detail? Were intervening factors described?	Y	Y	Y	Y	Y	Y	Y	Y	Y	Y	Y
7. Were outcomes or condition or status of interest clearly defined and the measurements valid and reliable?	Y	Y	Y	Y	Y	Y	Y	Y	Y	Y	Y
8. Was the statistical analysis appropriate for the study design and type of outcome indicators?	Y	Y	Y	Y	Y	Y	Y	Unclear	Y	Y	Y
9. Are conclusions supported by results with biases and limitations taken into consideration?	Y	Y	N	Y	Y	Y	N	N	Y	Y	Y
10. Is bias due to study’s funding or sponsorship unlikely?	Y	Y	Y	Y	Y	Y	Y	N	Y	Y	Y
**Overall Rating**^**b**^	**Positive +**	**Positive +**	**Positive +**	**Positive +**	**Positive +**	**Positive +**	**Positive +**	**Neutral Ø**	**Neutral Ø**	**Positive +**	**Positive +**

^a^The Academy of Nutrition and Dietetics Evidence Analysis Library (EAL) and Quality Criteria Checklist was used as the appraisal tools

^b^*Abbreviations*: *NA* Not Applicable. Positive (+) = most of the answers to the validity questions are “Yes” (including criteria 2, 3, 6, and 7 and at least one additional “Yes”). Neutral (Ø) = the answers to the validity criteria questions 2, 3, 6, and 7 do not indicate that study is exceptionally strong. Negative (−) = most (six or more) of the answers to the validity questions are “No”

### Data analysis

Qualitative analyses were carried out and results were presented narratively. For the qualitative analysis, difference in measures between NAFLD and control groups or pre- and post- in prospective studies and change between groups where appropriate, were reported, depending on the analysis reported for individual studies. Data were considered statistically significant if the reported *p*-value was < 0.05. Due to the heterogeneity of interventions, and measured outcomes, a meta-analysis was not possible.

## Results

### Study selection

The literature search process is shown in Fig. 1. The search strategy resulted in 1893 articles, of which 1229 remained after duplicates were removed. From this, 1135 articles were deemed ineligible as a result of title and abstract screening. Ninety-four studies were eligible for full-text screening and 84 were excluded for the following reasons: non-NAFLD population (*n* = 36), abstract only (*n* = 29), no cognitive outcome(s) (*n* = 15), duplicates (*n* = 2), and not available online (*n* = 2). One additional study was added through manual search of references (*n* = 1); there were no clinical trials found and thus, 11 observational studies were included in this systematic review.

### Study characteristics

The data extracted from the included 11 studies are presented in Table 1**.** All studies were of observational design; five were case-control [21, 24, 30, 31, 33], five were cross-sectional [22, 27–29, 32], and one was a cohort study [23]. The articles were published between 1984 and 2019. The studies were conducted in the United States, Turkey, Japan, Serbia, United Kingdom, Italy and Spain, and included a total of 7978 participants aged between 37 and 70 years (mean 51 years). The risk of bias assessment for each study is reported in Table 2. Nine articles received a positive quality rating [21–24, 27–30, 32], and two articles received a neutral quality rating [31, 33]; indicating majority of the studies posed a low risk of bias. While the risk of bias tool applied does not assess publication bias there appears to be a combination of positive and negative results in the included studies. The cognitive abilities and associated neuropsychological tests measured in each study are summarised in Table 3**.**

**Table 3:** Cognitive abilities and neuropsychological tests used in assessment in included studies^a^

Cognitive abilities	Neuropsychological tests^b^	Cognitive impairment seen in NAFLD	No difference seen in NAFLD	Study
**General Cognition**	Montreal Cognitive Assessment test *Serbian version*	+		Filipovic et al. 2018 [21]
	Montreal Cognitive Assessment *Turkish version*	+		Celikbilek et al. 2018 [22]
	Mini Mental State Examination	+		Tuttolomondo et al. 2018 [33]
	Cognitive Failures Questionnaire	+		Elliott et al. 2013 [23]
**Reasoning**	Raven’s Progressive Matrices		–	Tarter et al. 1984 [30]
**Mental speed, Attention and Psychomotor speed**	Digit Symbol Substitution Test	+		Weinstein et al. 2018 [27]
	MoCA-TR Attention: Sustained attention task		–	Celikbilek et al. 2018 [22]
	MoCA-TR Attention: A serial subtraction task		–	Celikbilek et al. 2018 [22]
	MoCA-TR Attention: Digits forward and backward tasks		–	Celikbilek et al. 2018 [22]
	Digit span forward		–	Tarter et al. 1984 [30]
	Digit span backward		–	Tarter et al. 1984 [30]
	Mental control		–	Tarter et al. 1984 [30]
	Purdue Pegboard	+		Tarter et al. 1984 [30]
	Stary Tracing		–	Tarter et al. 1984 [30]
	Symbol Digit Substitution Test	+		Seo et al. 2016 [32]
	The Simple Reaction Time Test	+		Seo et al. 2016 [32]
	Symbol Digit Modalities Test	+		Tarter et al. 1984 [30]
	The number connection test A		–	Felipo et al. 2012 [24]
	The number connection test B		–	Felipo et al. 2012 [24]
	The Serial Dotting Test		–	Felipo et al. 2012 [24]
**Memory and learning**	The Wechsler Memory Scale (verbal and visual)		–	Weinstein et al. 2019 [29]
	Logical memory		–	Tarter et al. 1984 [30]
	Figural memory		–	Tarter et al. 1984 [30]
	Paired associates		–	Tarter et al. 1984 [30]
	Supraspan - Weschler Memory Scale		–	Tarter et al. 1984 [30]
	Supraspan Learning Test	+		Tarter et al. 1987 [31]
	Digit Span Test		–	Tarter et al. 1987 [31]
	Benton Visual Retention Test	+		Tarter et al. 1987 [31]
	Rey-Osterreith Complex Figure Test		–	Tarter et al. 1987 [31]
	Brown-Peterson Test		–	Tarter et al. 1987 [31]
	The Serial Digit Learning Test	+		Seo et al. 2016 [32]
**Language**	Fluency		–	Tarter et al. 1984 [30]
	Confrontation Naming		–	Tarter et al. 1984 [30]
	Responsive Naming		–	Tarter et al. 1984 [30]
	Peabody Picture Intelligence Test		–	Tarter et al. 1984 [30]
	A Verbal Fluency Task	+		Takahashi et al. 2017 [28]
	The Token Test		–	Tarter et al. 1984 [30]
**Visuospatial perception**	MoCA-TR Visuospatial abilities: Clock drawing	+		Celikbilek et al. 2018 [22]
	MoCA-SR visuoconstructive: Draw a cube and a clock in 11:10 position of clock hands	+		Filipovic et al. 2018 [21]
	MoCA-TR Visuospatial abilities: Three-dimensional cube-copying task	+		Celikbilek et al. 2018 [22]
	Hooper Visual Organization Test		–	Weinstein et al. 2019 [29]
	Block Design		–	Tarter et al. 1984 [30]
	Tactual Performance	+		Tarter et al. 1984 [30]
	The Line Tracing Test		–	Felipo et al. 2012 [24]
**Ideas, abstraction, figural creations and mental flexibility**	The Animal Fluency Test	+		Weinstein et al. 2018 [27]
	Trail Making	+		Tarter et al. 1984 [30]
	MoCA-TR Executive functioning abilities: Trail Making B task	+		Celikbilek et al. 2018 [22]
	MoCA-TR Executive functioning abilities: Phonemic fluency task	+		Celikbilek et al. 2018 [22]
	MoCA-TR Executive functioning abilities: Two-item verbal abstraction task	+		Celikbilek et al. 2018 [22]
	Time to complete trail-making B minus time to complete trail-making A test	+		Weinstein et al. 2019 [29]
	The Similarities Test	+		Weinstein et al. 2019 [29]

^a^*Abbreviations*: *MOCA-TR* Montreal Cognitive Assessment Turkish, *MOCA-SR* Montreal Cognitive Assessment Serbian, *NAFLD* Non-alcoholic fatty liver disease, *NASH* Non-alcoholic steatohepatitis, *MMSE* Mini mental state examination, *T2DM* Type 2 diabetes mellitus, *CFQ* Cognitive failures questionnaire, *VFT* Verbal fluency task

^b^Plus (+) = impaired cognitive function observed in NAFLD for specific cognitive test. Negative (−) = no association or similar association observed in NAFLD for specific cognitive test

### Cognitive abilities

#### General cognition

Four studies including two case-control [21, 33], one cross-sectional [22] and one cohort study [23] investigated the associations between general cognitive performance and NAFLD using multiple neuropsychological tests. All four studies reported that individuals with NAFLD had significantly lower general cognitive function, measured with the Serbian [21] and the Turkish [22] MOCA (*n* = 76 and *n* = 213 respectively), MMSE (*n* = 163) [33], and the cognitive symptoms questionnaire (CFQ) (*n* = 431) [23].

#### Reasoning

One case-control study conducted in the US including 40 adults with a mean age of 41 years utilised the Raven’s Progressive Matrices to evaluate reasoning and found no significant difference between those with or without NAFLD [30].

#### Mental speed, attention and psychomotor speed

Five studies including three cross-sectional [22, 27, 32] and two case-control studies [24, 30] reported on the mental speed, attention and psychomotor speed. Overall, three studies indicated poorer performance in this cognitive domain in those with NAFLD [27, 30, 32], with one additional study indicating only those with the more progressed state of the disease, NASH, having poorer cognitive outcomes [24]. Two of these studies were cross-sectional studies conducted in the US and included 1102 and 4472 participants with mean ages of 69 and 41 years, respectively [27, 32]. Only one of the five included studies in this domain indicated that NAFLD or NASH was not associated with cognitive performance. One study reported that individuals with NASH had evidence of cognitive decline [22, 24]. Collectively, it appears that mental speed, attention and psychomotor speed in the majority (three out of five studies) was negatively influenced in individuals with NAFLD.

#### Memory and learning

Five studies including three case-control [22, 30, 31] and two cross-sectional studies [29, 32] utilised multiple neuropsychological tests to report on the memory and learning domain. Two observational studies (one case-control and a cross-sectional study) reported lower memory and learning test scores (Supraspan Learning test and Benton Visual Retention test and Serial Digit Learning test) in adults with NAFLD [31, 32]. Conversely, another three studies that used the Wechsler Memory Scale [27, 32] or the Delayed Recall Memory test (MoCA-TR) [22] did not observe significant difference in logical and figural memory among those with NAFLD. Collectively, the limited data available assessing memory and learning in those with NAFLD is conflicting and inconclusive.

#### Language

Two studies including one cross-sectional [28] and one case-control [30] examined the effects of NAFLD on the language domain of cognitive performance. One cross-sectional study in Japan (*n* = 39) reported significantly lower scores in the Verbal Fluency Task (VFL) among individuals with NAFLD [28]. On the other hand, another study in the US (*n* = 40) did not find significant group differences using the VFT, the Confrontation Naming task, and the Peabody Picture Intelligence test and Token test [30]. However, this latter study used a Crohn’s Disease control group. It is also difficult to compare the findings between studies due to the different cognitive function tests used. Overall, the number of studies and participants included in this cognitive domain is limited, include small sample sizes, and the findings are conflicting and thus inconclusive.

#### Visuospatial perception

Five studies including two cross-sectional [22, 29] and three case-control studies [21, 24, 30] reported on the visuospatial perception cognitive domain. Three of the five studies found poorer visuospatial perception scores, measured with MOCA [21, 22] or Tactual Performance task [30], in individuals with NAFLD. Conversely, no significant group differences were found in three studies for visuospatial perception as assessed using the Hooper Visual Organisation test [29], Block Design task [30] and Line Tracing test were used [24]. Together, these observations indicate that NAFLD may be associated with lower visuospatial perception and cognitive impairment.

#### Ideas, abstraction, figural creations and mental flexibility

Four studies including three cross-sectional [22, 27, 29] and one case-control [30] observed differences in the Ideas, Abstraction, Figural creations and Mental flexibility domain. Three studies (one case-control and two cross-sectional) reported significantly higher scores in the Trail Making task, indicating cognitive impairment, in individuals with NAFLD [22, 29, 30]. Another cross-sectional study that used the Animal Fluency test also observed cognitive decline (lower scores) in those with NAFLD [27]. Individuals with NAFLD also had poorer abstract reasoning skills in two studies, as measured by the Phenomic Fluency, Two-item Verbal Abstraction and Similarities test [22, 29]. In total, all available studies consistently reported that NAFLD was associated with poorer ideas, abstraction, figural creations and mental flexibility.

## Discussion

This systematic review, which is the first to examine the association between NAFLD and cognitive function, included 11 observational studies with 7978 participants across five countries. Based on the current literature available, the findings indicate that NAFLD is likely associated with poorer cognitive function across a number of domains. Specifically, three out of seven domains assessed in this review indicated there was evidence of poor cognitive performance in participants with NAFLD, including ‘general cognition’, ‘mental speed, attention and psychomotor speed’, and ‘ideas, abstraction, figural creations and mental flexibility’. The remaining cognitive domains (reasoning, memory and learning, language, visuospatial perception) produced conflicting and thus inconclusive findings potentially due to the limited number of studies and heterogenous designs and methodologies (e.g. study populations and cognitive tests).

The findings from this review indicating that NAFLD, the ‘hepatic manifestation’ of the metabolic syndrome, was associated with cognitive decline is in line with other literature indicating that metabolic syndrome and its components are strongly implicated in cognitive decline [35]. The reason why only three of the seven cognitive domains explored in this review were more likely to be impacted by NAFLD may relate to differences in the characteristics of the studies included. As all studies included the assessment of multiple cognitive domains, it is unlikely results showing cognitive decline with NAFLD were due to study design or sample size in the respective studies. Studies that investigate cognitive decline typically focus on older adults as this is when cognition is most sensitive to change [36, 37]. What was noted however was that only one of the 11 studies in this review included older adults with a mean age above 65 years and the overall mean age of participants in this review was middle aged [27]. Therefore, there was heterogeneity in the timing and rate of cognitive decline in different cognitive measures based on age and this may explain the mixed findings in terms of the link with only three of the seven cognitive domains assessed in this review. There are known disparate effects on cognition with numerous cognitive domains exhibiting decline such as memory and fluid cognition, while others are preserved with age such as language or vocabulary [38]. Declines in ‘general cognition’, ‘mental speed, attention and psychomotor speed’, and ‘ideas, abstraction, figural creations and mental flexibility’ may be explained by the fact that cognitive tasks requiring verbal fluency, processing or transforming information to make a decision, working memory and executive functioning are particularly sensitive to changes with age [35]. This decline is worsened with age, but the fact that this review demonstrated a decline in cognition which was more pronounced in NAFLD in individuals aged 37-61 years suggests that this condition may contribute to early onset cognitive decline particularly in certain domains.

There are numerous potential underlying mechanisms that may explain the possible early onset of cognitive decline with NAFLD. These include insulin resistance and progressive lipid deposition in the liver in NAFLD, comorbidities which are highly prevalent in middle aged populations and have been shown to increase peripheral hyperinsulinemia, lipid peroxidation, and systematic inflammatory damage to brain cells [38]. Obesity, T2DM and the MetS co-exist with NAFLD and are all driven by inflammation and oxidation, which contribute to impaired vascular function, and subsequently poorer cognitive function [39]. Furthermore, emerging evidence suggests that NAFLD poses an additional risk for dysbiosis by disrupting the gut brain axis and thus may also deteriorate cognition in individuals with this disease [40]. Liver diseases especially NAFLD and its more progressed form, NASH, can lead to elevated ammonia levels (also known as hyperammonemia) [41, 42], and when combined with inflammation this can lead to cognitive impairment [24]. This is supported by the only study in this review that assessed NAFLD severity and demonstrated that participants with NASH showed significant cognitive impairment compared to those with only simple steatosis (NAFLD) [24]. Therefore, NAFLD co-existing with multiple co-morbidities (e.g. chronic diseases, hyperinsulinemia, systemic inflammation and extrahepatic change to the central nervous system) and/or more progressed NAFLD, namely NASH, theoretically will exacerbate cognitive impairment. In part support of this notion, there is evidence in middle-aged adults showing that cognitive decline is associated with the presence of other comorbidities such as adiposity [40]. The findings from this review shows some early evidence that this may be the case for NAFLD and cognition, although more research is needed to confirm this relationship.

This review contains several strengths including a comprehensive and systematic search in multiple databases and achieving an overall positive Risk of Bias assessment score (Table 2). Furthermore, this review provides early evidence on the possible association between NAFLD and cognition across various domains. All of the studies included measured cognitive function using validated diagnostic criteria, including a variety of standardised neuropsychological tests such as MoCA, MMSE and CFQ. This review was also robust in that we pooled and discussed studies based on cognitive domains using a previously established method [26]. However, the review had several limitations such as the small number of studies and participant numbers included and an absence of clinical trials to demonstrate causation. All studies were observational in design and predominantly case-control and cross-sectional. A further limitation was the heterogeneous diagnostic tools, with unknown cross-comparability used to measure cognitive function, making the comparison of research findings difficult. This limitation has also been raised in previous reviews, where there is an urgent need for consensus on using standard cognitive assessments [44, 45]. In addition, due to the heterogeneity of populations, with regard to co-morbidities and severity of NAFLD and tools to assess cognition amongst studies and the scarcity of literature reporting on the relationship between NAFLD and cognitive function, a meta-analysis could not be conducted. Finally, there is a known association between cognition and other chronic conditions such as diabetes and cardiovascular disease and therefore without well designed studies that can control for these cardiometabolic conditions it is difficult to deduce what the role of NAFLD is specifically, outside of the cluster of metabolic conditions.

## Conclusion

In conclusion, this review of 11 studies indicates that there is an association between NAFLD and lower cognitive performance. Particularly, that young and middle-aged adults with NAFLD had poorer cognitive function across several domains, including ‘general cognition’, ‘mental speed, attention and psychomotor speed’, and ‘ideas, abstraction, figural creations and mental flexibility’. This suggests that NAFLD in mid-life may accelerate cognitive decline in certain domains, particularly those that aren’t preserved with older age. However, prospective, adequately powered longitudinal studies that used valid and sensitive tools are needed to confirm the association between NAFLD and cognition in the future. Future studies should also consider standard tools to enable comparison of results between studies, in order to promote a better understanding of the relationship between NAFLD and cognition, and as practical tools to identify those at risk of cognitive decline.

## Data Availability

Detailed findings of the selection process of included and excluded articles are available upon request.

## Abbreviations

ALD: Alcoholic liver disease
AFT: Animal fluency test
ALT: Alanine aminotransferase
ALP: Alkaline phosphatase
AST: Aspartate aminotransferase
BMI: Body mass index
BVRT: Benton visual retention test
CFQ: Cognitive Failures Questionnaire
CVD: Cardiovascular disease
DSST: The digit symbol substitution test
DST: Digit symbol test
HVOT: Hooper visual organisation test
HTN: Hypertension
LTT: Line tracing test
MoCA-TR: Montreal cognitive assessment Turkish version
MoCA-SR: Montreal cognitive assessment Serbian version
MCI: Mild cognitive impairment
MetS: Metabolic syndrome
NAFLD: Non-alcoholic fatty liver disease
NASH: Non-alcoholic steatosis
NCTA: Number connection test A
NCTB: Number connection test B
PRISMA: Preferred reporting items for systematic reviews and meta-analysis
SIM: Similarities test
SRRT: Simple reaction time test
SDST: Symbol digit substitution test
SDT: Serial dotting test
SDLT: Serial digit learning test
T2DM: Type 2 diabetes mellitus
VFT: Verbal fluency task
